# Electrical property comparison and charge transmission in p-type double gate and single gate junctionless accumulation transistor fabricated by AFM nanolithography

**DOI:** 10.1186/1556-276X-7-381

**Published:** 2012-07-11

**Authors:** Arash Dehzangi, A Makarimi Abdullah, Farhad Larki, Sabar D Hutagalung, Elias B Saion, Mohd N Hamidon, Jumiah Hassan, Yadollah Gharayebi

**Affiliations:** 1 Department of Physics, Faculty of Science, Universiti Putra Malaysia, Serdang, Selangor 43400, Malaysia; 2 School of Materials and Mineral Resources Engineering, Universiti Sains Malaysia, Nibong Tebal, Penang, 14300, Malaysia; 3 Functional Devices Laboratory, Institute of Advanced Technology, Universiti Putra Malaysia, Serdang, Selangor 43400, Malaysia; 4 Department of Chemistry, Islamic Azad University, Behbahan Branch University Street, Behbahan, 6361713198, Iran

**Keywords:** Atomic force microscopy, Junctionless transistors, Local anodic oxidation, Silicon-on-insulator, Double gate, Single gate junctionless silicon nanowire transistor

## Abstract

The junctionless nanowire transistor is a promising alternative for a new generation of nanotransistors. In this letter the atomic force microscopy nanolithography with two wet etching processes was implemented to fabricate simple structures as double gate and single gate junctionless silicon nanowire transistor on low doped p-type silicon-on-insulator wafer. The etching process was developed and optimized in the present work compared to our previous works. The output, transfer characteristics and drain conductance of both structures were compared. The trend for both devices found to be the same but differences in subthreshold swing, ‘on/off’ ratio, and threshold voltage were observed. The devices are ‘on’ state when performing as the pinch off devices. The positive gate voltage shows pinch off effect, while the negative gate voltage was unable to make a significant effect on drain current. The charge transmission in devices is also investigated in simple model according to a junctionless transistor principal.

## Background

The aggressive trend of scaling transistors requires a new and more effective device to catch up with this rapid trend for modern transistors. Several innovations in fabrication process such as high κ dielectrics [1], metal gate electrodes [2], stressors [3], and new transistor architectures based on silicon-on-insulator (SOI) such as Fin field-effect transistors (FETs) [4], multigate FETs [5], omega-gate FETs [6], gate-all-around FETs [7], or developed non-epitaxial raised metal Schottky source drain [8] have been introduced. In the recent years, junctionless transistors (JLTs) appeared to be the promising alternative for new generation of transistors [9]. All existing transistors contain semiconductor junctions. Contemporary transistors with ultrasmall size need ultrasharp doping concentration gradients in junctions. The doping must switch from ultra high concentration n-type to p-type along the very small area in size of some nanometers, which imposes severe limitations on the processing thermal budget and requires the development of costly millisecond annealing techniques. In JLT the doping concentration in the channel source and drain is uniform with high concentration profile for the channel in order to have a reasonable amount of current flow when the device is turned on [10]. The lack of doping concentration gradients provides the smaller size and cancels the need for costly ultrafast annealing techniques. In the last two years, the research in JLTs was focused in design and property [11-13], simulation [14-16], high temperature performance [17], and new fabrication method with higher mobility and better performance [18-20].

The principle of atomic force microscopy (AFM) nanolithography, using local anodic oxidation (LAO) on SOI, has been described for the first time by Snow and Campbell et al. [21,22], and they astutely expanded AFM nanolithography for fabrication of nanostructures. Ionica et al. [23] have remarkably reported the electrical characteristics of the devices made by AFM nanolithography. In the recent years, some new works have been performed to improve the method of AFM nanolithography [24,25]. However, the lack of sufficient explanation or interpretation for the behavior of these structures is still an interesting issue and worth for further investigation. In fact, fabrication of nanotransistors by AFM nanolithography with similar structure has been developed with prominent result in the last decade, but recent rising of the JLTs theory and fabrication can bring up the AFM nanolithography as the extra alternative. We already reported the fabrication of the p-type single gate (SG) JLT device with a simple structure, low doping concentration, and no gate oxide layer [26-29]. The most important advantage of AFM nanolithography is that it impedes damage of the crystalline structure of silicon due to highly energetic electrons which are normally introduced to the structure by techniques such as electron beam lithography.

In this paper we report the fabrication of a double gate structure with improved method by implementing the advantages of AFM nanolithography in contact mode with a simple structure. We used the SOI technology to ensure a very sharp interface top silicon layer-silicon dioxide and used buried oxide layer as an etch stop layer during fabrication. Low doping concentration p-type SOI was used in order to have less scattering effect and low ‘off’ current. Also, the electrical property of both double gate (DG) and single gate JLT will be compared; the charge transmission, according to the JLTs’ principal with the glance of accumulation mode transistors function, will be investigated.

## Methods

### Device fabrication

**Figure 1:**
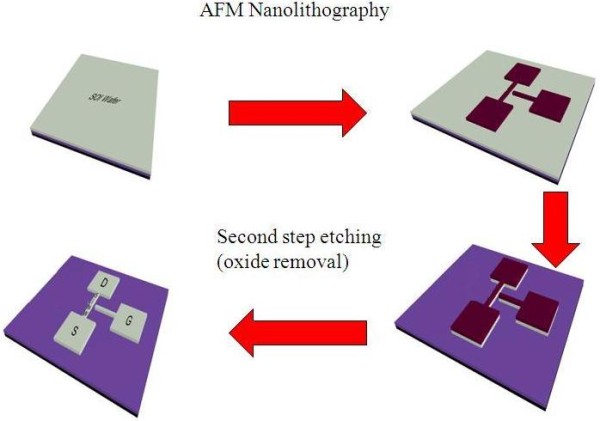
Schematic of SG-JLSNWT fabrication steps.

The AFM nanolithography process was performed by using scanning probe microscope machine (SPI3800N/4000, SII Nanotechnology Inc., Chiba-shi, Chiba, Japan) in contact mode. Low doped (10^15^ cm^−3^) p-type (100) SOI wafer was prepared using Unibond™ (Unibond International Ltd., Uxbridge, Middlesex, UK) processed with a 145-nm buried oxide (BOX) thickness, 90-nm top Si layer thickness, and a resistivity *ρ* of 13.5 to 22.5 Ω cm used as the substrate [30]. Prior to use, the SOI wafer was cut into small sizes (1 × 1 cm), cleaned by modified standard Radio Corporation of America cleaning process and then dipped in hydrofluoric acid (HF) (1% water solution) for 30 s in order to replace the Si-O bonds by low energy Si-H bonds. After sample preparation, AFM nanolithography with LAO method was applied to provide etching of the stop layer on top of SOI substrate. Finally, KOH anisotropic etching and HF oxide removal etching completed the fabrication of device (Figure 1). 

### Local anodic oxidation

**Figure 2:**
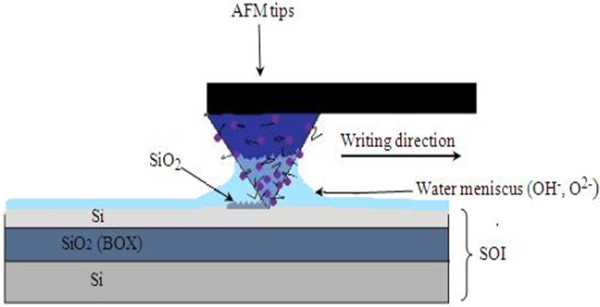
Schematic presentation of LAO process on SOI sample surface.

An AFM tip (Cr/Pt conductive coating) was used to draw oxide patterns on top of the SOI substrate. The hydrogen atoms can be locally removed on the surface of the substrate with AFM when a negative tip voltage provides a local oxidation by means of field-enhanced oxidation process. The voltage pulse was applied to form a liquid bridge between the tip and the sample; meanwhile, another voltage was applied to the silicon substrate to induce nano-oxidation, and the absorbed water layer on the surface provided the required electrolyte under this ambient condition. During the oxidation, the force reference was −0.1 N, also the writing speed, scan speed, and applied tip voltage were held at 0.5 μm/s, 1.0 μm/s, and 9 V, respectively (all parameters were optimized). All mentioned parameters with the relative humidity percentage of 65% to 68% provided the thickness of 3 nm for oxide layer, which is an acceptable range, and the patterned structure is well-shaped. The air ambient humidity is essential to achieve the oxidation [31,32]. In Figure 2, the LAO process is schematically shown. 

**Figure 3:**
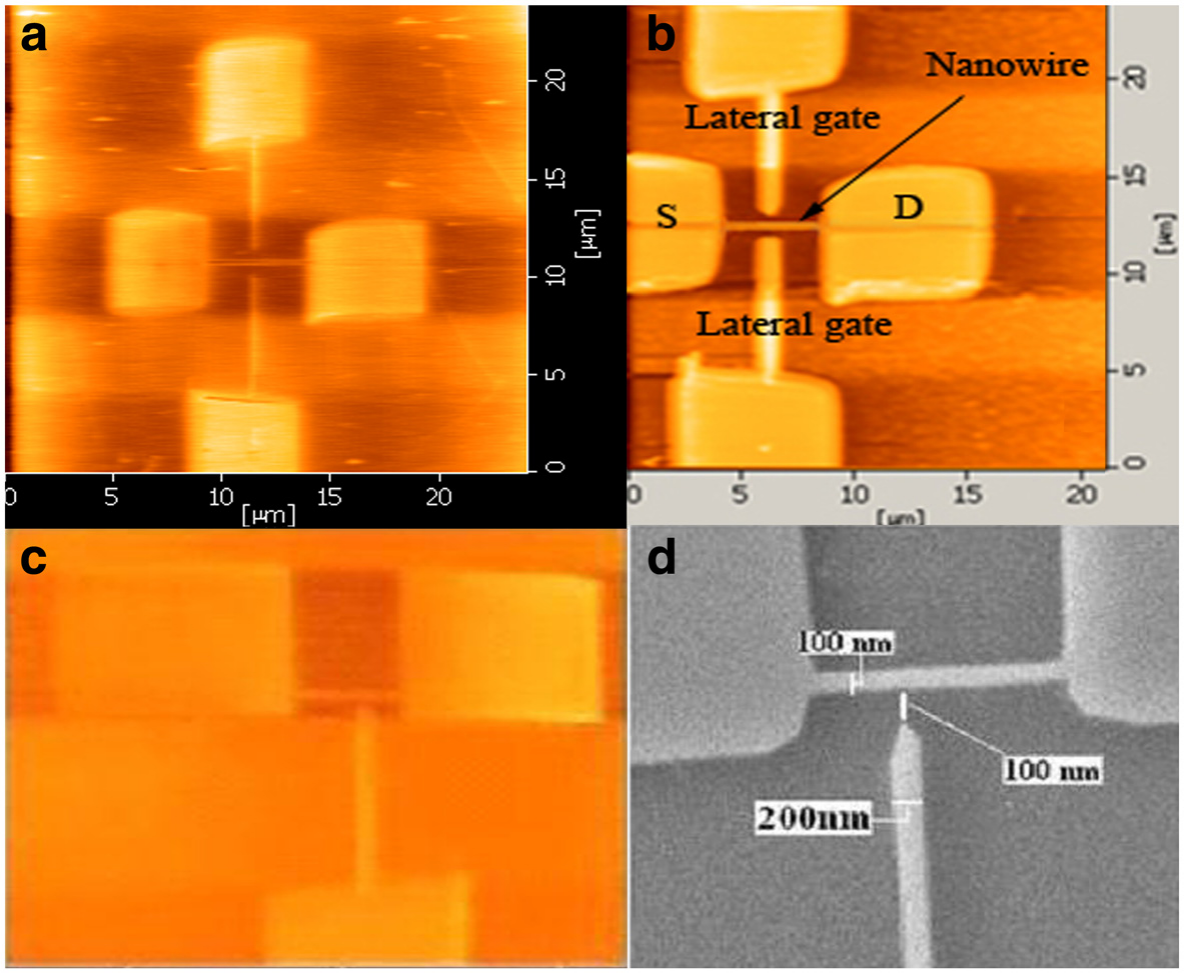
AFM and SEM images of DG (a,b) and SG (c,d) JLTs after LAO and etching process.

Figure 3a,b,c,d shows the DG and SG structures after the LAO with the best gate symmetry and the smallest reproducible dimensions we had achieved. In the SG structure the nanowire moved towards the side gate to avoid of leakage current appeared in the previous work [27]. 

### Wet etching process

KOH wet etching is a very significant part in the fabrication of SG and DGJLT. In fact, having contamination, ill-etched, or over etching structures were hardly avoidable in wet etching which, accordingly, the accuracy and precaution are important. To remove the undesired Si area, KOH was used as an etchant. The KOH concentration also affects the quality of the etched surface. Referring to the previous reports in KOH wet etching [33-37], we used a 30 wt.% KOH solution, saturated with isopropyl alcohol (IPA) at 63°C to remove all the non-protected silicon areas. IPA was used in this work as initiator to improve the cleaning process providing smooth surface. IPA reduces the etch rate, hence improving the surface roughness and making the etching process more controllable. The best optimized condition was the solution of 30 wt.% KOH with 10 vol.% IPA for wet etching at 63°C, immersing time for 20 s, and stirring at 600 rpm. The stirring of the solution is to ensure the uniformity of the etching process.

The final structure was obtained by removing the oxide layer using HF acid (Figure 3b,d). In fact, several models have been proposed for the silicon anisotropic etching mechanism in aqueous KOH, which we have chosen as the method by considering of the crystallographic planes for a cubic crystal. In a cubic crystal, the (110) plane is normal to the diagonal of a surface plane, and the (111) plane is normal to a volume diagonal. For the atoms located on the (100) plane, they have two dangling bonds and two bonds remaining in the crystal. Like in our case, when a (100) plane is exposed by the etching solution, OH^−^ can attach to the dangling bonds and loosen the other bonds, so they can break easily. The KOH etching of (100)-oriented silicon provides V-shaped grooves [33].

**Figure 4:**
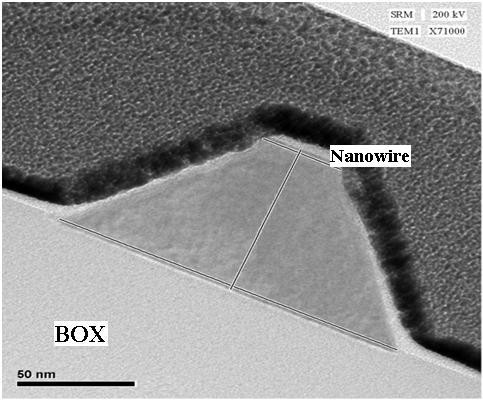
High magnifications TEM micrograph of etch depth profile to show the dimension of the nanowire.

In Figure 4, the high magnification transmission electron microscopy (TEM) micrograph of the etch depth for nanowire profile is shown for another sample. The adjacent gate is not shown in the picture. The SOI with 90 nm thickness of the Si layer makes the whole structure, after etching with the thickness of 90 nm. Both SG and DG structures have 100 nm for the channel width, 200 nm for the channel length, and 4 μm for the distance between the source and the drain. The gap between the gate and the channel for both structures were 100 nm (Figure 3). The electrical connections were provided by two pads considered as source and drain with the gate work function of 5.12 eV. The Si thickness of the whole structure including the channel, gate pad(s), source and drain contacts were 90 nm with the same p-type doping concentration (10^15^).

## Results and discussion

### Results

**Figure 5:**
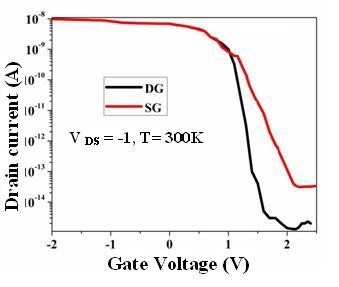
Transfer characterization graph for DG and SG JLT.

The electrical characterization of the transistor was characterized by semiconductor parameter analyzer (Lakeshore, Desert Cryogenics Agilent HP 4156 C, Agilent Technologies, Santa Clara, CA, USA). Figure 5 shows the transfer characteristic for DG and SGJLT. The pinch off effect can be seen due to positive lateral gate applying on the channel. The subthreshold swing (SS) and on/off ratio for double gate junctionless transistor (DGJLT) were 10^6^ and 100 mV/decade, respectively, and for SGJLT were 10^5^ and 167 mV/decade respectively. By increasing the positive gate voltage, the current dropped. This indicates that the device required positive gate voltage to be turned off. The pinch off effect for both devices is recognizable which *off* state occurred in +1.5 and +2.5 V in DG and SG structures, respectively. The result for output characteristic shows that the drain current (*I*_D_) does not significantly increase with the negative increase of gate voltage (not shown), unlike the conventional p-type channel metal-oxide-semiconductor field-effect transistor (MOSFETs). Also, high and positive threshold voltage (+1.2 V for SG and +0.8 V for DG) suggests that the device is in an *on* state. It indicates that the transistor was in the *on* state with zero gate voltage.

**Figure 6:**
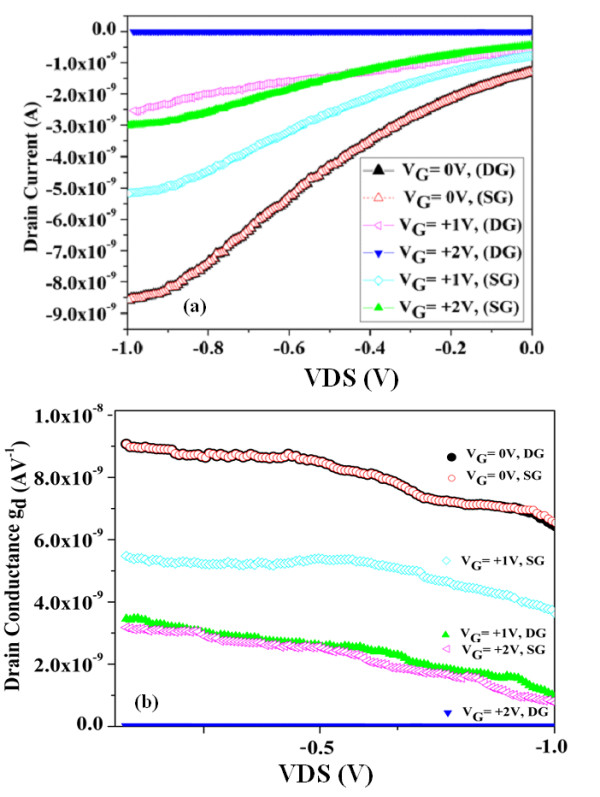
Output characteristic (a) and drain conductance (b) for DG and SGJLT.

The *I*_D_*V*_DS_ characteristics for SG and DG structures are shown in Figure 6 for positive gate voltage. Low current is due to the low doping concentration profile (10^15^ cm^−3^) for the channel, which is lower than reported current value of the high doping concentration profile (5 × 10^19^ cm^−3^) JLTs. The MOSFETs or JLTs with high doping concentration mostly suffered with the high scattering effect or threshold voltage variation. Low channel doping can improve field-effect mobility for improved transconductance and drive current and decrease the scattering effect and thresholdvoltage variations [38]. It can also provide low current in an *off* state; however, low off current is achievable by increasing the gate work function values. The electrical characteristics of the devices have the same trend compared to the reported cases fabricated by AFM nanolithography with nearly similar structure [21,39,40]. In fact, in none of the reported cases, the devices were used as the pinch off device. Normally, high doping SOI was implemented and was never checked to use in a reverse bias to investigate the pinch off effect. Also in our work, we do not have remarkable increase in the current due to the negative gate voltage, while some previous works have shown a higher rate for increasing the current under the gate voltage (mostly positive voltage for the n-type case), and also a higher current value (due to the higher doping concentration). 

In Figure 6a, we can recognize the effect of the gate on channel in a DG structure which is more effective than SG due to the asymmetry of SG. In the DG structure the pinch off effect was achieved in *V*_G_ = +2, while this value cannot provide the same current value in SG. This required higher voltage to approach pinch off effect in SG structure which was in *V*_G_ = +3 V (Figure 5). Figure 6b shows the drain conductance for SG and DG structures under the different gate voltage. By increasing the gate voltage, the drain conductance for both structures will be decreased. For comparing DG structure to SG device, we have a more effective gate voltage in the channel approaching the pinch off effect (*off* state) with lowest drain conductance, which is consistent to the output characteristics and our expectation about the DG structure. The trend for drain conductance is the same with MOSFETs [41] and JLTs [42], yet the slope is smaller here which can be explained by low doping concentration and current value.

### Subthreshold swing

Although the SS value is relatively higher than the best value in single crystal silicon devices [15] and recent JLTs [20,43,44], it is still comparable with the lowest reported value of vertical silicon nanowire array devices [45]. In general, the degradation of SS is due to the increase in the interface state density, decrease of oxide capacitance, and increase in the doping concentration of metal oxide silicon transistor’s channel [46]. However, in our work the interface state density probably cannot play an important role since we have only one interface with the channel (channel/BOX interface), and the current value is low. The most important reason for higher SS value in our case could be explained by the lack of oxide layer between the gate and the channel. It lacks the fixed potential drop in cross section of the nanowire (perpendicular to the current flow), which is necessary for inducing sufficient potential to change current linearly with the gate voltage [44]. In SGJLT device, the asymmetry of the gate location provides higher SS value compare to DGJLT device with the symmetric gate locations.

### Model description

In recent reports on experimental JLTs [18,47], we did not encounter any case of *on* state condition under the zero gate voltage due to having an opposite doping concentration for the gate and the channel, unless for the simulation cases and for very small gate lengths [48]. The charge transmission in DG and SGJLT operates quite differently from the conventional MOSFETs and also slightly different from the JLT description in recent literature. The devices are working in *on* state for nonzero *V*_DS_ and *V*_G_ = 0 V. The reason can arise from the fact that the field effects from the different work function of the gate and channel cannot cause the device to be turned off at *V*_G_ = 0 V due to the same doping concentration of the channel and gate contact, and no oxide layer for the gate.

Basically, regardless of the gate work function difference between the gate electrode and channel, JLTs are ‘gated resistor’ which is in the *on* state at *V*_G_ = 0 V [16]. According to the JLT’s principal, when the device is turned on, it approaches the flat band condition. It basically behaves as a resistor, and the electric field perpendicular to the current flow is equal to zero in the ‘bulk’ channel. In fact, as the advantage of our fabrication method, the AFM lithography keeps the surface and the body of the upper Si layer of the SOI intact and untouched. So we expect to find more bulk property, for example, higher mobility and less surface scattering effect for the channel under the gate.

**Figure 7:**
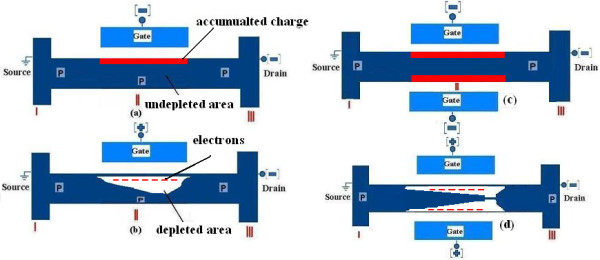
Schematic operations (a,b) of the SG and DG (c,d) for positive and negative gate voltage.

Immediately after applying *V*_SD_, the device goes to an *on* state. The *on* current is controlled by the semiconductor doping concentration and not by the gate capacitance. The operation of devices is outlined in Figure 7, and the three regions I, II, and III are denoted. The structures are a gated resistor turned off by depleting the channel (region II), when essential positive gate voltage is applied. It will be turned off based on the pinch off effect principle, when *V*_G_ provides a sufficiently large barrier in the gating region; the highest depletion occurs near to the drain side of the channel due to the stronger electric field in the drain side (Figure 7b,d). Figure 7a,c schematically show the devices in the *on* state. In this condition, the subthreshold current flows by increasing the *V*_DS_ until the saturation current will be reached at region II, even for *V*_G_ = 0 V. Since the system is in *on* state from *V*_G_ = 0, one can say that the threshold voltage is shifted into the positive voltage, and the neutral wire is instantaneously shaped when the bias is applied to the source/drain contacts (Figure 7a,c). That is the reason one can claim that the devices are already in flatband condition like the pinch off transistors [49,50]. In the *on* state condition, the holes concentration in the channel increases, and the neutral or undepleted channel forms between the source and the drain until the peak of the holes concentration in the channel reaches the doping concentration N_A_. 

**Figure 8:**
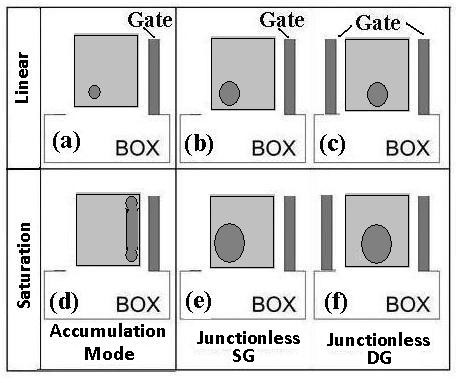
**Profile view of holes location transmission path in different devices (modified from**[10]**).**

In Figure 8, the schematic profile view of holes location transmission path and comparison of accumulation mode device with SG and DG device are shown. For the DGJLT the neutral wire locates in the center of the channel and close to the bottom, as shown in Figure 8, c and f. It is worth to mention that in SGJLT, the neutral or depleted wire will be formed not exactly in the center. Due to the specific shape of the device and having only one interface with BOX at the bottom, the neutral wire must be formed near to the bottom of the channel and away from the side gate sidewall (Figure 8, b and e). By further increasing the *V*_DS_ in the *on* state, the depletion will be starting near to the drain due to high electric field in this area in region III, and this is the reason for having saturation for the current [51]. The high electric field in the drain gives rise to the full depletion in nanowire near to the drain area acting as a buffer against the high electric field in the drain, which accordingly, will lead the current to be saturated. 

However, by negatively raising the gate voltage, it is probable to have a little increasing of the current due to some accumulated charges, which were injected from the source (region I) to the channel (red color areas in Figure 7). The drain current mainly flows through a bulk channel. An additional small conduction likely originated from a lightly accumulated channel in sidewalls facing the gates, when the gate voltage is large enough. The influence of the gate on the channel is not very effective to induce an accumulation mode due to the device configuration, low doping concentration, and the lack of oxide layer between the gate and the channel. Accordingly, increasing the gate voltage cannot help to make an effective accumulation layer and we do not expect to have the accumulation mode for high gate voltage. Normally in high doping JLTs in *on* state, after increasing the gate voltage, the device is able to be converted into the accumulation mode with significant increasing of the current (mostly is not desired to reach) [9,47]. Actually, another reason that we interpret the devices as JLT and not in accumulation mode is the ineffective negative increasing gate voltage on the channel.

In the accumulation mode, in *on* state condition, the subthreshold current flows through the bulk of the device near the center of the nanowire just like the JLTs (Figure 7a). But the magnitude of this current is less than ten percent of the whole current achievable. By increasing the gate voltage, the majority of the holes are confined in inversion layers at the sidewalls, with marked peaks at the corners (Figure 7d). In the reported and high doping JLTs, we can increase the gate voltage in order to have accumulation charge and raise the current after reaching the flatband. But still, the largest part of the current is due to bulk conduction. The formation of a surface accumulation channel is also observed at high *V*_G_. In comparison to the trigate FETs, Fin FETs, gate-all-around (GAA) FETs or reported JLTs in which there were more interfaces with the gate oxide layer or BOX and more current values after increasing the negative gate voltage for p-type channels. Here, we only have one interface between the channel and the BOX in order to provide charge accumulation. Accordingly, the increasing negative gate voltage is not able to produce more current compare to the trigate FETs, Fin FETs, GAA FETs, or reported JLTs. For our SG and DGJLTs as gated resistors, when the device is turned on, these essentially behave as a resistor, and the drain current is controlled by regions I and II and doping concentration. For linear region *I*_D_ could be given approximately by [47]:

(1) $$I_{D}\approx q\mu N_{A}\frac{TW}{L}V_{\mathrm{DS}}.$$

Where N_A_ is the semiconductor doping concentration, *μ* is the effective mobility, *q* is the electron charge; *T*, *W*, and *L* are the thickness, width, and the length of the channel respectively. Equation 1 was first time suggested by Colinge [9] for JLTs and also by Fonash et al. [52] who also suggested the similar equation before Colinge’s group about the accumulation mode of unipolar Si nanowire transistors. This equation points out that *I*_D_ is controlled by the doping concentration N_A_, and not by the gate capacitance per area C. We believe that the *I*_D_ equation in our case would be very similar to Equation 1. For high doping concentration cases, which were mostly considered in literatures for JLTs, Equation 1 is suggesting for linear region. In our case, considering the *on* state device, low concentration profile for the p-type material, and also the effect of the fins at the side of the channel to the source and the drain contacts, we suggest the same equation, unless, for the *V*_DS_ we have effective voltage for the channel *V*_Ch_, which is obeying the $\left|V_{\text{Ch}}\right|<\left|V_{\text{DS}}\right|$. Then, we have

(2) $$I_{\text{D}}\approx q\mu N_{\text{A}}\frac{TW}{L}V_{\text{Ch}}.$$

In the *on* state condition, for a given *V*_DS_, the electric field from the source to the negatively biased drain must be significantly small (nearly zero) in the neutral wire at the center of the channel. In the linear region we expect that the negative charge in region I (Figure 7), which is adjacent to the area of I/II interface, should be gathered. This charge in the p-type material can only come from depletion in the channel in linear region.

In our case to enter the saturation region from linear region and since the device is trying to reach the saturation condition, we propose that we would have the condition at which the effective channel voltage become fixed at *V*_Ch_^Sat^. Then, further increases in *V*_DS,_ take place across the channel region and causes the negative charge (electrons) accumulation at the two sides of the channel near the source and the drain interfaces with channel and also in $\left|V_{\text{Ch}}^{\text{Sat}}\right|<\left|V_{\text{DS}}\right|.$ Considering the Colinge et.al suggestion [47] for saturation region, we have

(3) $$I_{\text{DSat}}\approx q\mu N_{A}\frac{TW}{2L}{\left(V_{\text{Ch}}^{\text{Sat}}\right)}^{2}.$$

This equation can be compared with the general expression of drain current for conventional MOSFETs in the saturation region or even in the accumulation mode [53]. In addition, because of the presence of ohmic contacts for the majority carriers and their location, which is away from the channel edges, we will not have any ambipolar behavior. Unfortunately, the transistors showed leakage through the gate electrode when gate voltages exceeded −3 V. However, the device worked was acceptable for gate voltages smaller than −3 V and gave us some information to confirm our simple model.

## Conclusions

The DG and SGJLT were fabricated by AFM-LAO nanolithography on low doped p-type SOI, followed by two improved wet etching process. We do not have a conventional situation for above the threshold voltage and channel saturation, since the devices are gated resistor and *on* state pinch off transistor. Then negative *V*_G_ cannot provide the accumulation in channel, but the pinch off occurs alike in a regular junctionless field-effect transistor. The output and transfer characteristic comparison of DG and SG structures were shown and the simple model according to the JLT principal.

## Competing interest

The authors declare that they have no competing interests.

## Authors’ contributions

AD designed and carried out the experimental work, conducted basic characterizations of the sample, analyzed all the data, the model description, and wrote the manuscript. AMA performed the TEM observations and participated in characterization. FL conceived of the study and participated in the experimental work, design, and coordination. SDH participated in the sequence alignment and provided the AFM and SPA instruments. EBS and MNH supervised the research work. JH critically revised the manuscript and YG participated in sequence alignment. All authors read and approved the final manuscript.
